# SARS-CoV-2 Vaccine Improved Hemostasis of a Patient with Protein S Deficiency: A Case Report

**DOI:** 10.3390/ijms251910717

**Published:** 2024-10-05

**Authors:** Mohammad A. Mohammad, Alaa Malik, Lekha Thangada, Diana Polanía-Villanueva, Jovanny Zabaleta, Rinku Majumder

**Affiliations:** 1 Department of Interdisciplinary Oncology, Louisiana State University Health Sciences Center (LSUHSC), New Orleans, LA 70112, USA; mmoha4@lsuhsc.edu (M.A.M.); amali5@lsuhsc.edu (A.M.); lthang@lsuhsc.edu (L.T.); jzabal@lsuhsc.edu (J.Z.); 2 Stanley S. Scott Cancer Center, Louisiana State University Health Sciences Center, New Orleans, LA 70112, USA; dpolan@lsuhsc.edu

**Keywords:** protein S, COVID-19, protein S deficiency, Pfizer vaccine, COVID-19 vaccination, thrombosis

## Abstract

A 16-year-old patient, while an infant, incurred right-sided hemiparesis and had difficulty breast feeding. She was later diagnosed with a neonatal stroke and her genetic testing showed a missense mutation in her *PROS1* (Protein S) gene. Both her grandfather and father, but not her mother, had hereditary Protein S (PS) deficiency. The patient was not prescribed any mediation due to her young age but was frequently checked by her physician. The patient’s plasma was first collected at the age of 13, and the isolated plasma from the patient and her father were analyzed by aPTT, thrombin generation, and enzyme-linked immunosorbent assays. These analyses showed low PS activity and clotting time associated with the missense mutation in the *PROS1* gene. During the COVID-19 pandemic, the patient received her first Pfizer vaccination dose in 2021, followed by a booster dose in 2022. The plasma samples were collected 8 weeks post-immunization, after which her clotting parameters had improved for up to 6 months following vaccination. The patient’s plasma showed a significant reduction in thrombin generation and an improved aPTT clotting time. Mass spectrometry analysis revealed that her antithrombin-III level was significantly higher post-vaccination, and both thrombin and FXII levels were significantly lowered compared with her father. To our knowledge, this is the first report to document that COVID-19 vaccination can lower the risk of thrombosis in a patient with inherited thrombophilia. Although the effect was observed on a single mutation, it would be interesting to investigate the effect of COVID-19 vaccinations on other thrombophilia.

## 1. Introduction

A well-documented delicate balance exists between the procoagulant and anticoagulant proteins in the control of tissue hemostasis and homeostasis [1]. Disruption of this balance results in a spectrum of coagulation disorders [2]. For instance, Protein S (PS) is a key anticoagulant that has an essential function in the coagulation process by acting as a cofactor for Activated Protein C [3] and Tissue Factor Pathway Inhibitor [4]. Furthermore, we showed that PS directly binds FIXa, thereby inhibiting FIXa-mediated activation of FX and limiting subsequent thrombin formation [5].

Genetic and acquired PS deficiencies are extensively documented. Although the symptoms associated with acquired PS deficiency are temporary, genetic alterations in the *PROS1* gene can result in pulmonary embolism, thrombophilia, venous thromboembolism and defects in development of the vascular system [6]. An association between hypercoagulability and altered PS activity has been reported in conditions such as liver and kidney disease, pregnancy, the use of oral (estrogen-based) contraceptives, hypoxia, chemotherapy [7], and COVID-19 [8]. Patients affected by COVID-19 have a high risk of venous thromboembolism, and PS deficiency increases the risk of thrombosis by 20% for those patients [9]. Also, lower PS activities were detected in serum of COVID-19 patients, and it was found that PS served as a marker for recurrent stroke [10] and poor prognosis [11].

Although vaccines for COVID-19 provide protection against viral-associated diseases, venous thromboembolism is nevertheless a common symptom post-vaccination [12]. However, in this case study, we present a novel finding that an mRNA-based COVID-19 vaccine improved anticoagulation and limited thrombin generation in a 16-year-old patient with a PS deficiency who had a history of neonatal stroke.

## 2. Case Presentation

Sanger DNA sequencing was used to determine the cause of the patient’s stroke. We determined that both she and her father had a methionine-643 to threonine (M643T) missense mutation (rs750531364) within the *PROS1* gene on 3q11.1 (Table 1). Formstone et al. reported that this mutation increases the risk of thrombosis [13]. In addition, a variant in intron12 (rs1453447448) was also identified in the patient’s *PROS1* gene. This mutation overlaps 11 transcripts and causes the substitution of threonine to cysteine or alanine. However, no prior reports of this mutation have been documented.

**Figure 1:**
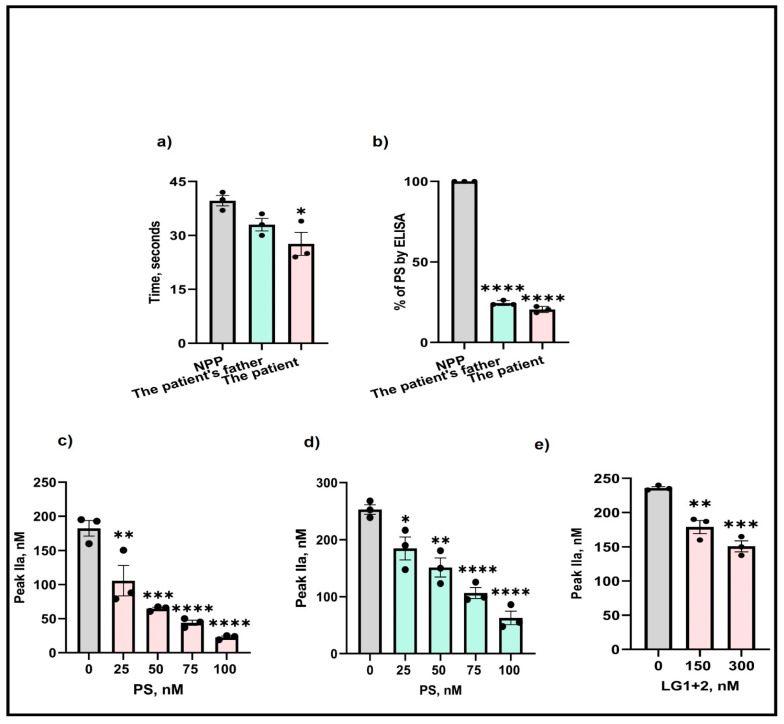
Effects of M643T mutation on PS determined by (**a**) aPTT, (**b**) ELISA, and thrombin formation produced by (**c**) the patient, (**d**) the patient’s father, and (**e**) in presence of added PS LG1 + 2 domains. The values shown are the mean ± SD of three independent experiments in triplicate. Statistical analyses are performed by One-way ANOVA * *p* < 0.05, ** *p*, < 0.05, *** *p* <0.0001, **** *p* <0.0001.

Next, to examine whether the M643T mutation affected the expression of PS, isolated plasma from the patient and her father were analyzed by aPTT and ELISA. Assessments of the clotting time in the patient’s plasma showed a 1.5-fold reduction in clotting time (Figure 1a) compared with the control (NPP—normal pooled plasma). Also, measurement of free PS by ELISA indicated an 80% and 74% reduction in the patient’s and her father’s plasma (Figure 1b), respectively.

To determine the effects of the M643T mutation on thrombin generation, isolated plasma from the patient and her father were supplemented with various concentrations (25–100 nM) of wild-type PS, and thrombin generation was measured. Before the addition of PS, the amount of thrombin produced in the PS-deficient patient’s plasma was 180 nM. However, supplementation with PS gradually decreased thrombin formation, reaching a significant 1.7-fold reduction (40 nM) after the addition of 100 nM PS (Figure 1c). Similar effects were observed for the father’s thrombin formation (Figure 1d).

The LG1 and LG2 domains of PS are important for binding to and inhibiting FIXa (our unpublished data). Therefore, we supplemented the plasmas with various LG1+2 concentrations (150–300 nM) and again assessed thrombin formation. The addition of LG1+2 to 300 nM resulted in a significant 1.2-fold reduction in the amount of thrombin produced, indicating the importance of these domains in the anticoagulant function of PS (Figure 1e).

**Figure 2:**
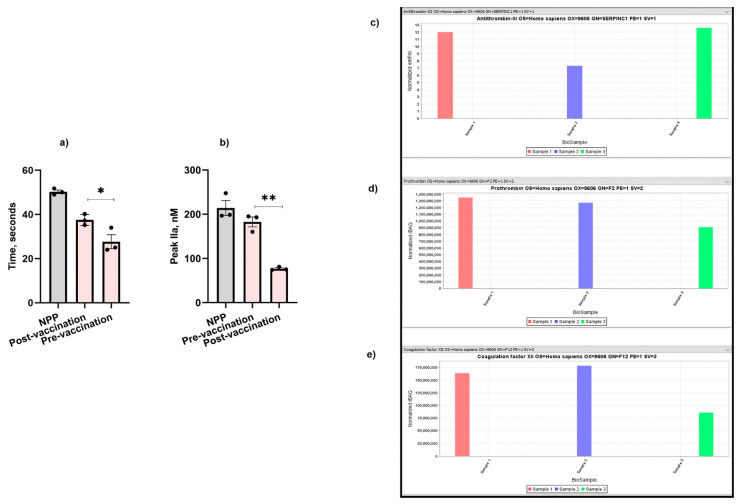
Effect of COVID-19 vaccination on the patient’s (**a**) aPTT, (**b**) TGA, (**c**) antithrombin-III, (**d**) thrombin, and (**e**) FXII. Sample 1: Control. Sample 2: The patient’s father. Sample 3: The patient. The values shown are the mean ± SD of three independent experiments in triplicate. Statistical analyses are performed by One-way ANOVA * *p* < 0.05, ** *p*, < 0.05.

Following COVID-19 vaccination (Pfizer, New York City, NY, USA), the patient’s clotting time was increased by 12% (Figure 2a) and her thrombin formation was also significantly reduced by 2.4-fold post-vaccination (Figure 2b) compared to pre-vaccination (Figure 1a,b). The patient experienced an increase in the level of antithrombin-III (Figure 2c) (normalized exponentially modified protein abundance index (emPAI): 12.5; normalized intensity Based Absolute Quantitation (iBAQ): 2,500,000,000) compared with her father who was also vaccinated (normalized emPAI: 7.3; iBAQ: 1,900,000,000) and the control sample (normalized emPAI: 12; normalized iBAQ: 2,100,000,000). The level of thrombin was also significantly reduced in the patient (normalized iBAQ: 900,000,000) compared with the thrombin level of her father (normalized iBAQ: 1,290,000,000) and the control sample (normalized iBAQ: 1,330,000,000) (Figure 2d). Similarly, FXII was significantly lower in the patient’s plasma (normalized iBAQ: 87,000,000) compared to her father (normalized iBAQ: 180,000,000) and the control (normalized iBAQ: 163,000,000) (Figure 2e).

## 3. Discussion

Protein S deficiency is an autosomal-inherited thrombophilia which manifests as venous or arterial thrombosis. The symptoms associated with PS deficiency can vary from asymptomatic to severe, depending on the type of mutation and the age of onset [14]. In early infancy, compound heterozygous or homozygous PS deficiency may result in purpura fulminans [15,16], and standard anticoagulation therapy is usually recommended throughout adulthood [14]. There are over 453 variants recorded worldwide as of the year 2000 [17]. Since then, more variants have been identified in the *PROS1* gene leading to various hereditary thrombophilia disorders [18]. In this case study, we identified several variants within the *PROS1* gene in both the patient and her father. Most importantly, we identified a missense mutation (Table 1), *rs750531364 c.1832T* > *C (p. Met611Thr),* whereby methionine is substituted by threonine at residue 643 and causing PS deficiency. This variant resides in the LG2 domain of PS near the LG1+2 junction where residues E435 and E437 are responsible for binding FIXa (unpublished data), thereby limiting thrombin formation. Because of this mutation, her free PS levels are low (Figure 1b) and therefore PS cannot exert its effect on FIXa, resulting in a faster clotting time (Figure 1a) and higher thrombin formation (Figure 1c).

Since the pandemic, inherited PS deficiency has not been linked to prognosis of COVID-19, although Mele et al. (2022) reported that PS deficiency exacerbated the thrombotic burden leading to the death of a patient [19]. Acquired PS deficiency has been attributed to hypercoagulability and the increased risk of thrombosis in COVID-19 patients [20]. Several studies have linked mRNA vaccines and thrombosis [21], and one of the earliest pulmonary embolism cases induced by an mRNA-based COVID-19 vaccine was recorded in South Korea [12]. The exact mechanism behind these observations is unclear. It has been suggested that the virus spike protein expressed by the mRNA vaccines promotes the release of pro-inflammatory proteins and the subsequent activation of coagulation proteins [22] or that the spike protein activates TAM (TYRO3, AXL and MER) signaling receptors, leading to immuno-thrombosis [23]. Studies have also documented that COVID-19 vaccinations can alter the expression profile of non-coding RNA (ncRNA) such as microRNAs (miRNA) and long ncRNAs (lncRNAs) [24,25,26,27,28,29], resulting in various outcomes ranging from thrombosis [30] to reducing the severity of COVID-19 [31]. For instance, it has been reported that the expression of miR-195-5p and PS positively correlate [32] and both can serve as biomarkers of disease severity [33,34].

Initially, we selected various pro- and anti-coagulant proteins for the mass spectrometry analysis. However, there is a lack of plasma and published studies reporting the effect of COVID-19 vaccines on the increased the levels of FXII, FXIII, prothrombin, and antithrombin [35]. Therefore, we also selected FXII, antithrombin, and prothrombin for further analysis. Surprisingly, post-vaccination, the patient’s clotting time was improved (Figure 2a), which was also reflected on the lower thrombin formation (Figure 2b) compared to pre-vaccination values (Figure 1a,b). We also observed a reduction in the levels of FXII (Figure 2e) and prothrombin (Figure 2d) in the patient’s plasma, whereas her antithrombin-III (Figure 2c) level was significantly higher. Therefore, it is possible that the activation of FXI by FXII is hindered within the blood vessel walls, thereby affecting the activation of downstream coagulation factors. It would be interesting to test whether Pfizer’s COVID-19 vaccine has a similar effect on PS-deficient individuals having other PS mutations, and it would also be interesting to test the relevance of this observation in other inherited thrombophilia disorders.

## Figures and Tables

**Table 1:** Genetic variants identified within the *PROS1* gene in the patient and her father.

Name	Chr	Position	ID	Ref	Alts	VarQual	Type	Gene Symbol	Transcript	Gene Section	Nt Change	AA Change	Genotype
The patient													
	3	93873725	rs9681204	T	G	228	3-prime UTR	PROS1	NM_001314077	Exon 16	c.520A>C	None	GG
	3	93877004	rs750531364	A	G	222	Missense	PROS1	NM_001314077	Exon 15	c.1928T>C	M643T	AG
	3	93886205	rs1453447448	T	C	5.34	Intron	PROS1	NM_001314077	Intron 12	c.1419+131A>G	None	TC
	3	93886282	rs8178649	A	G	222	Intron	PROS1	NM_001314077	Intron 12	c.1419+54T>C	None	AG
The patient’s father													
	3	93873725	rs9681204	T	G	225	3-prime UTR	PROS1	NM_001314077	Exon 16	c.520A>C	None	GG
	3	93874275	rs6123	T	C	222	Synonymous	PROS1	NM_001314077	Exon 16	c.2097A>G	None	TC
	3	93877004	rs750531364	A	G	222	Missense	PROS1	NM_001314077	Exon 15	c.1928T>C	M643T	AG
	3	93886282	rs8178649	A	G	228	Intron	PROS1	NM_001314077	Intron 12	c.1419+54T>C	None	GG
	3	93896513	.	C	CT	44.4	Intron indel	PROS1	NM_001314077	Intron 10	c.1061+63G>G	None	REF/ALT
Control 1													
	3	93873725	rs9681204	T	G	225	3-prime UTR	PROS1	NM_001314077	Exon 16	c.520A>C	None	GG
Control 2													
	3	93873725	rs9681204	T	G	228	3-prime UTR	PROS1	NM_001314077	Exon 16	c.520A>C	None	GG
	3	93900539	.	C	A	4.38	Intron	PROS1	NM_001314077	Intron 8	c.823+265G>T	None	AA

## Data Availability

Dataset available on request from the authors.
